# Supplementary material for: Linear ubiquitination induces NEMO phase separation to activate NF-κB signaling
Source: Life Sci Alliance. 2023 Jan 31;6(4):e202201607. doi: 10.26508/lsa.202201607 (PMC9889916; doi:10.26508/lsa.202201607)

1D

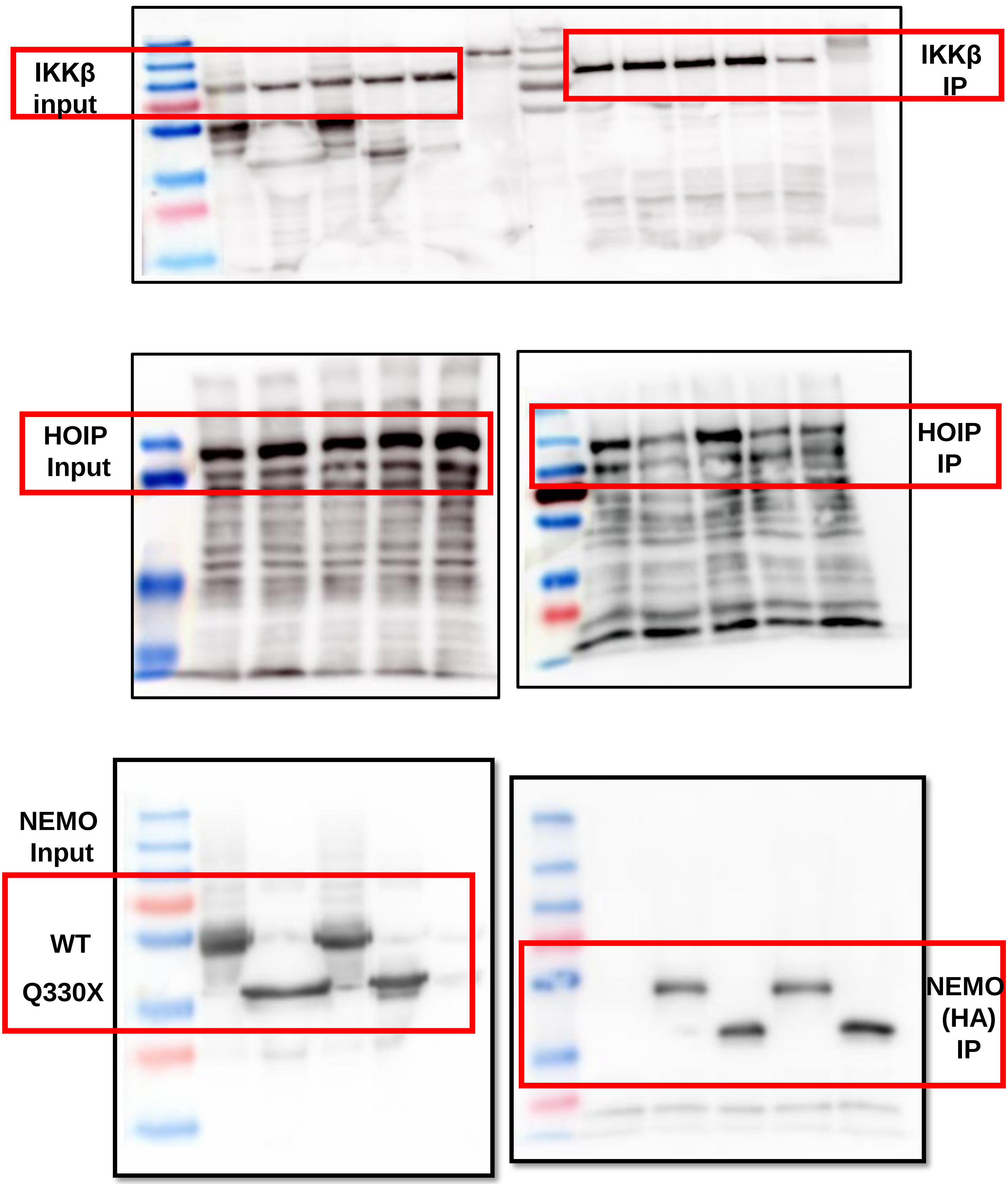
IKKβ input
IKKβ IP
HOIP Input
HOIP IP
NEMO Input
WT
Q330X
NEMO (HA) IP

1D

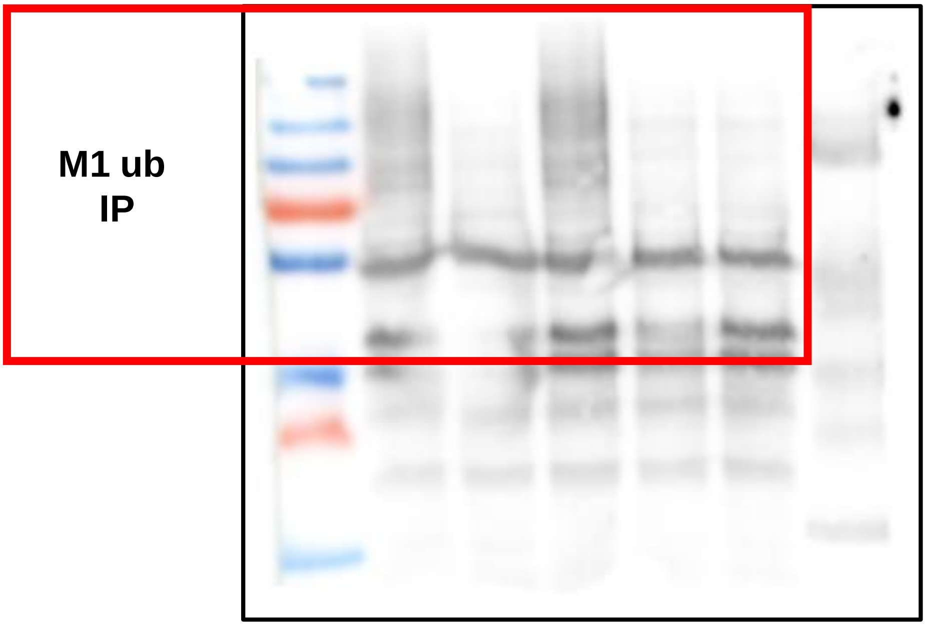

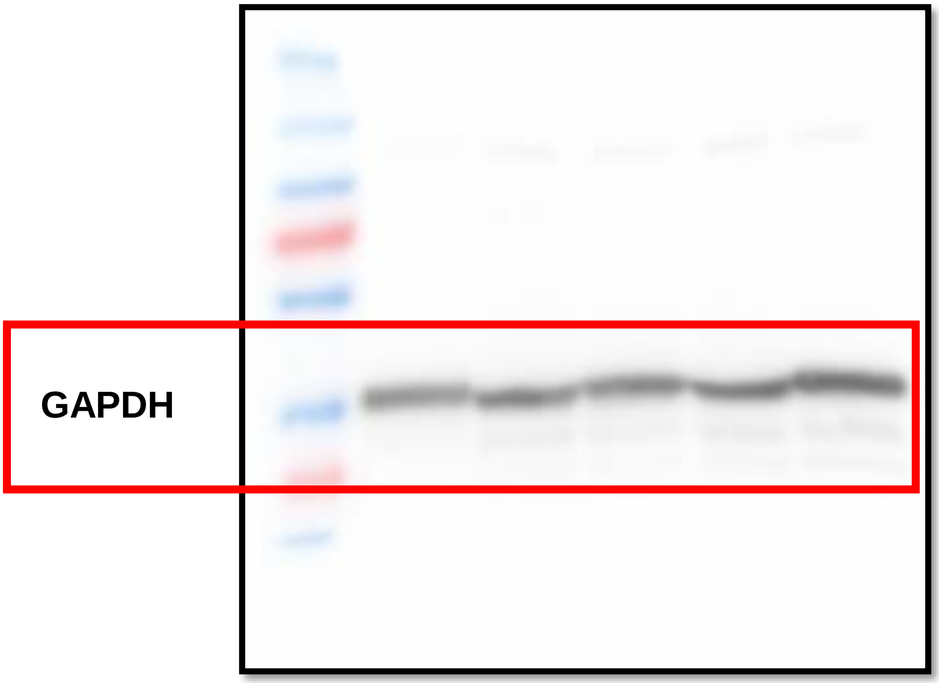

1C

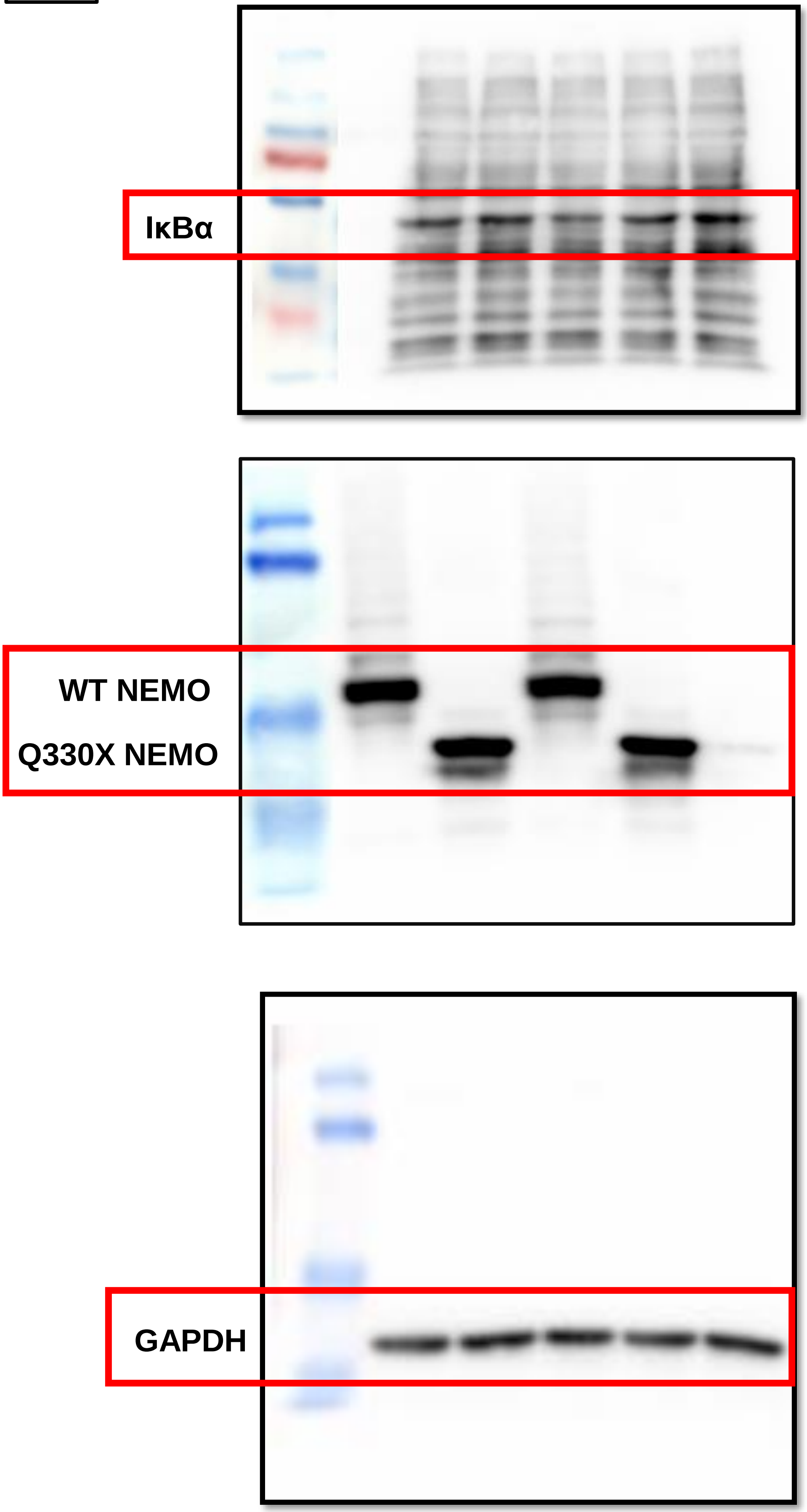

Supplement: Supplementary file 2 [file LSA-2022-01607_SdataF1.2.pdf]
