# Supplementary figures and images for: Linear ubiquitination induces NEMO phase separation to activate NF-κB signaling
Source: Life Sci Alliance. 2023 Jan 31;6(4):e202201607. doi: 10.26508/lsa.202201607 (PMC9889916; doi:10.26508/lsa.202201607)

2A

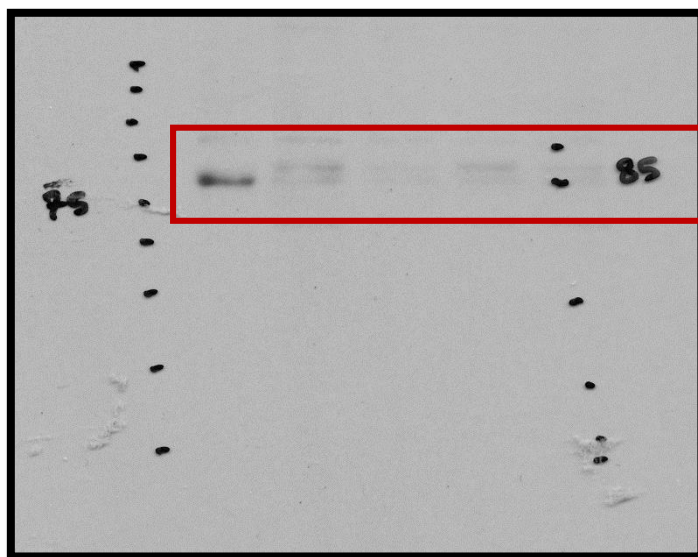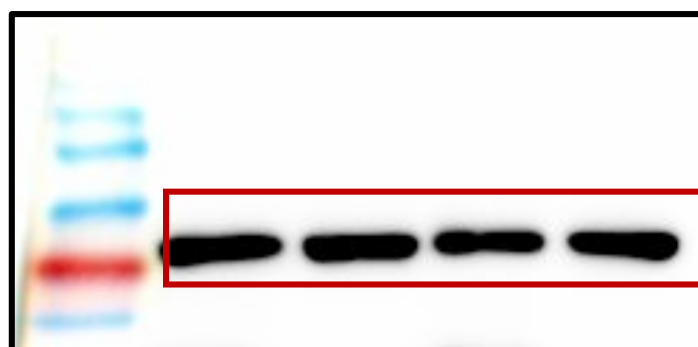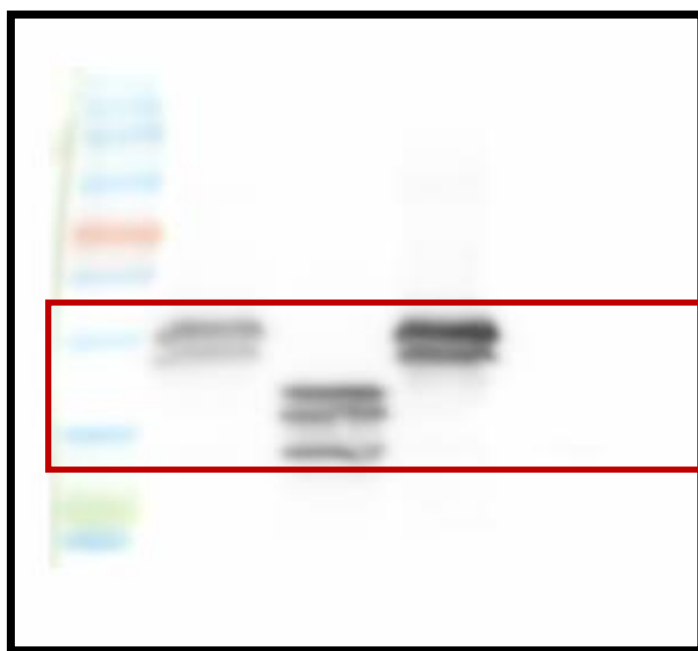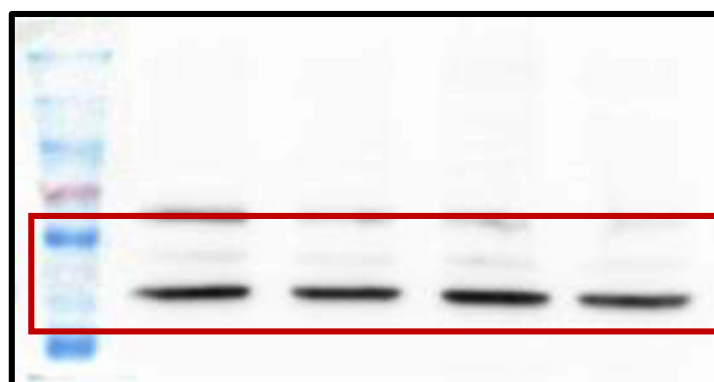

2B

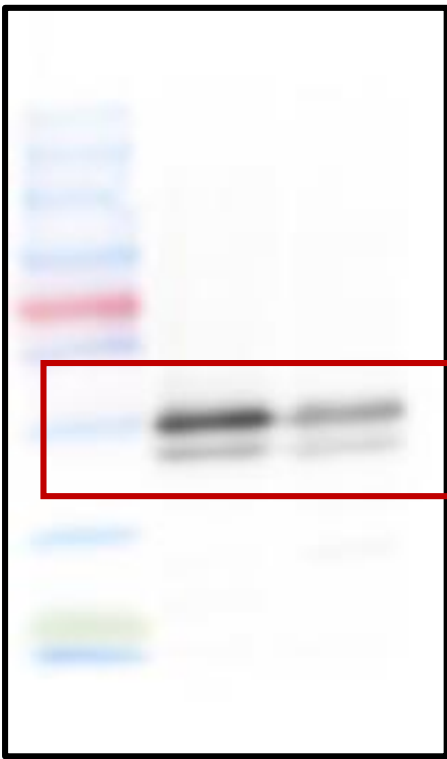

NEMO

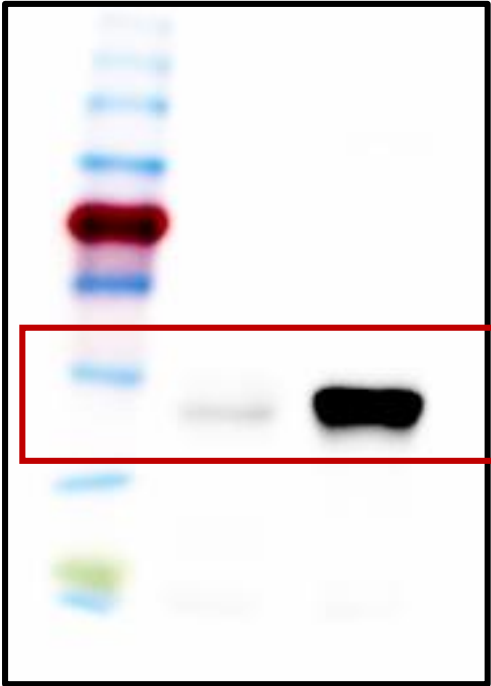

OTULIN

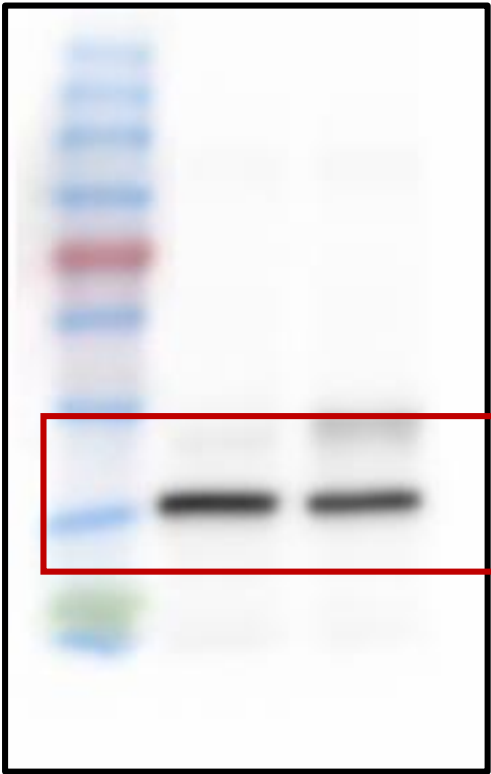

GAPDH

2C

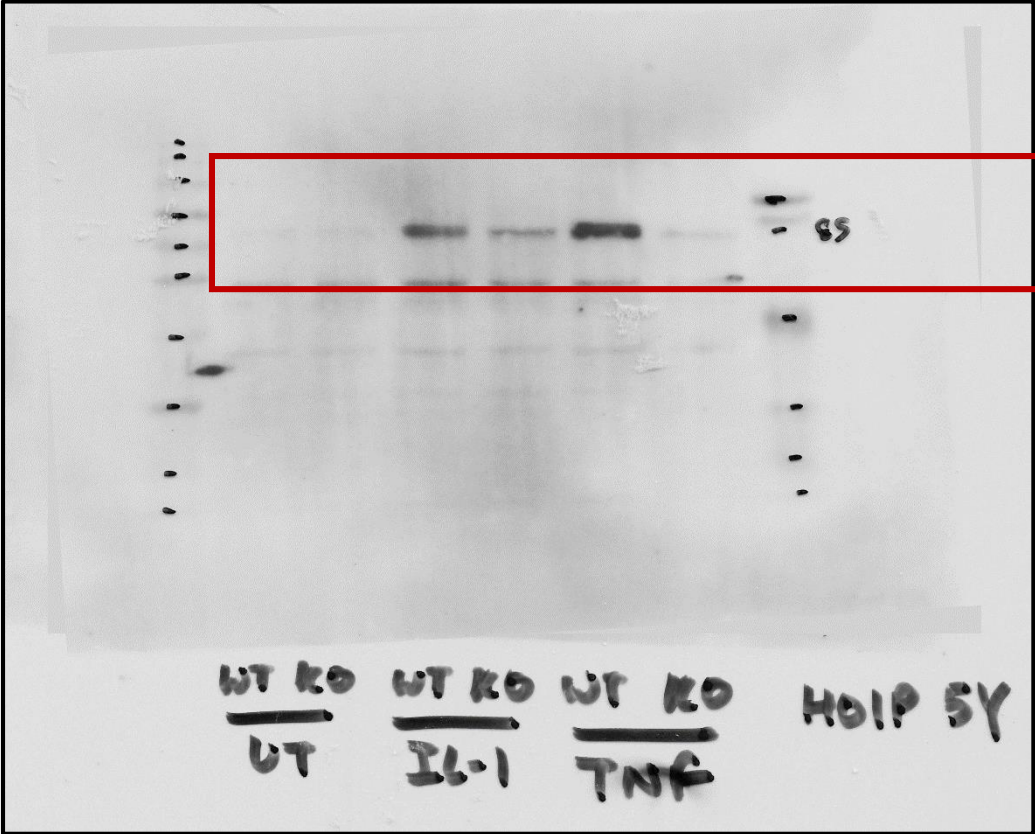

p-IKK

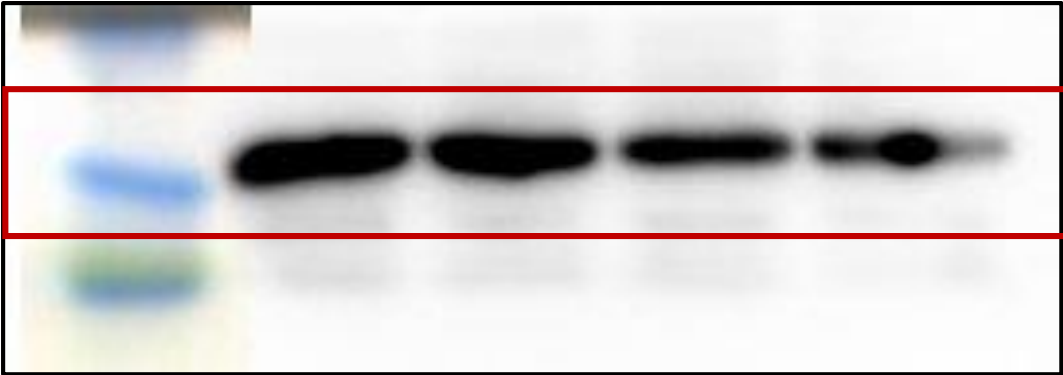

GAPDH

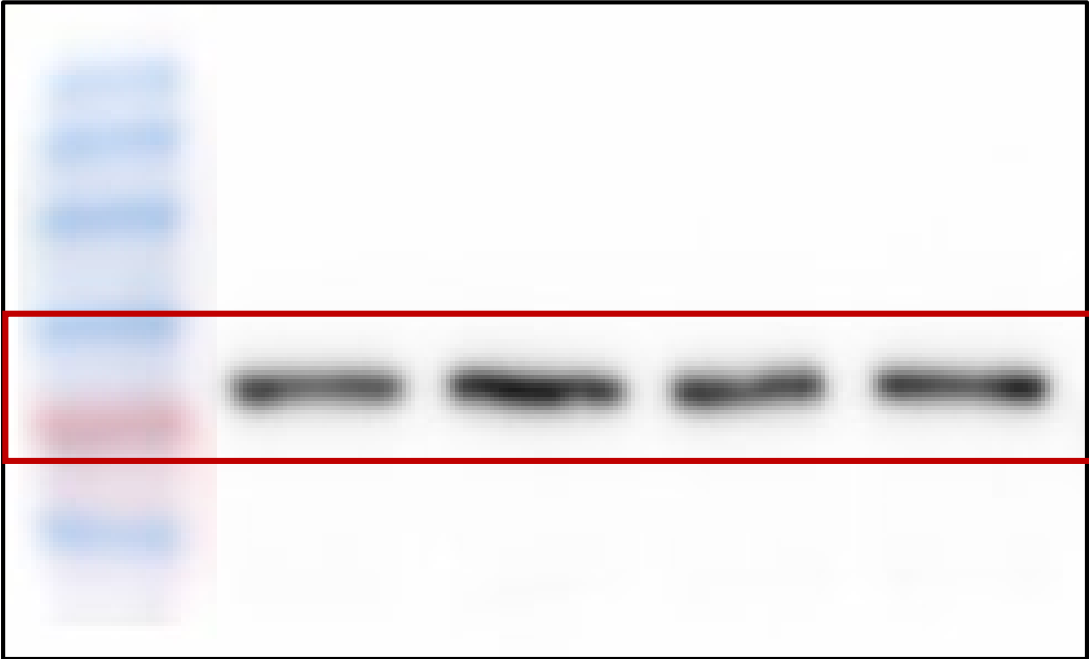

IKK

2C

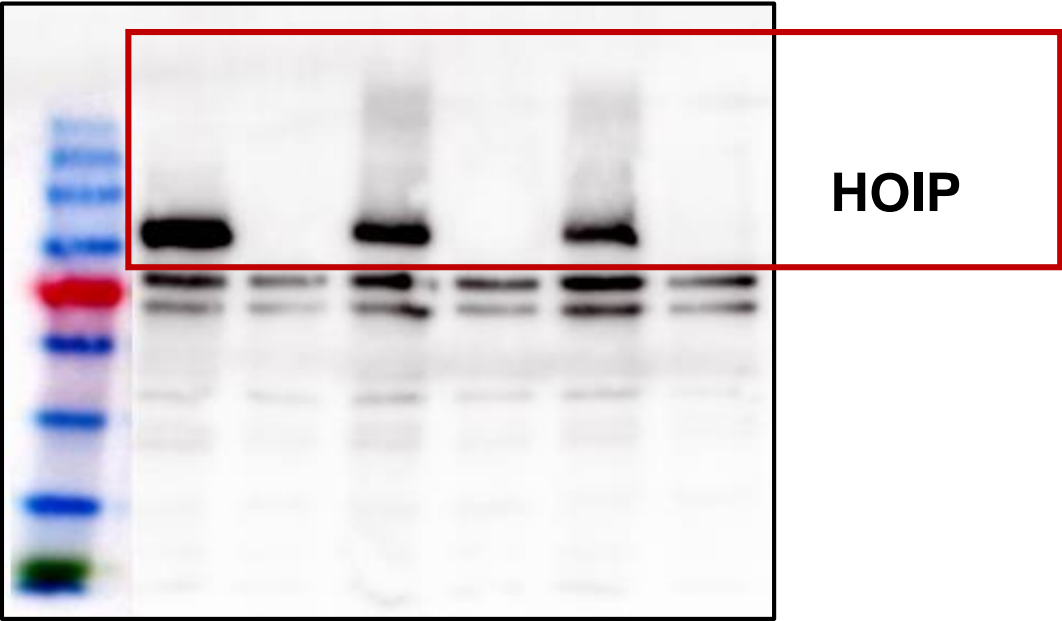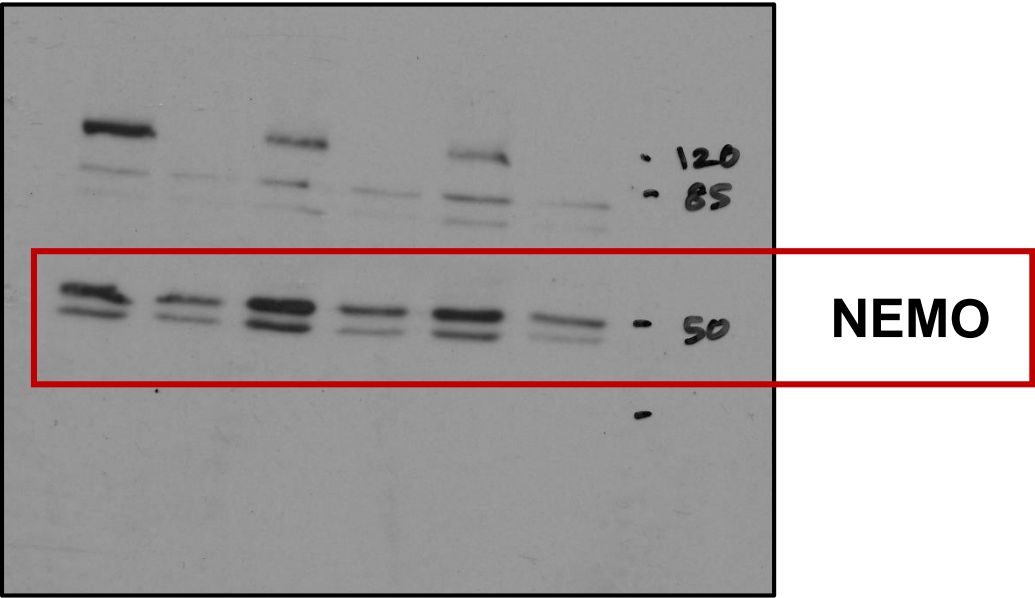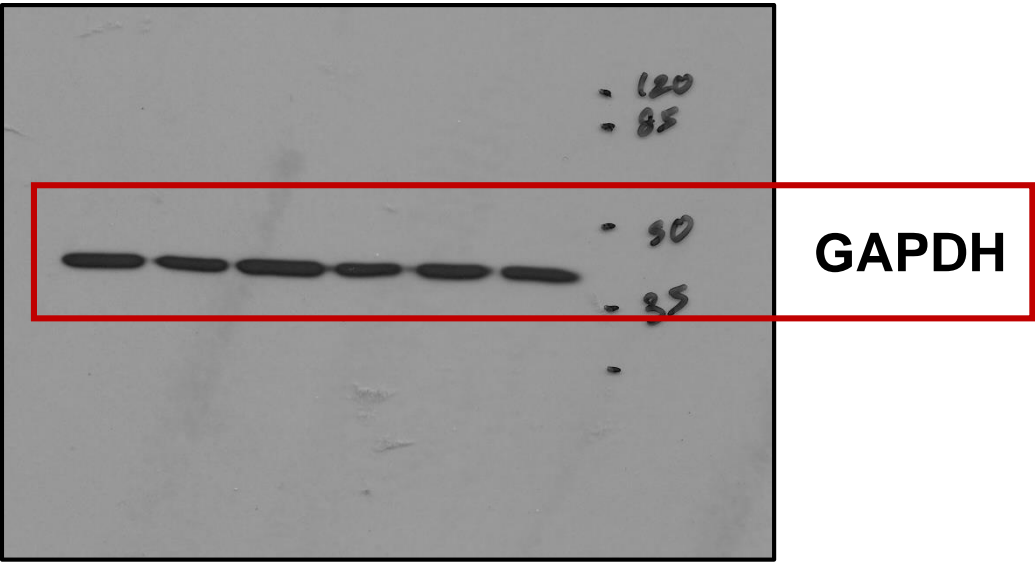

Supplement: Supplementary file 4 [file LSA-2022-01607_SdataF2.2.pdf]

3A

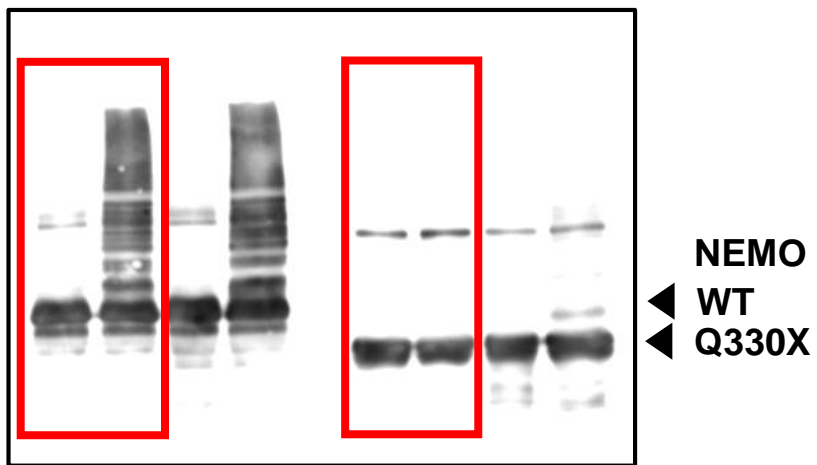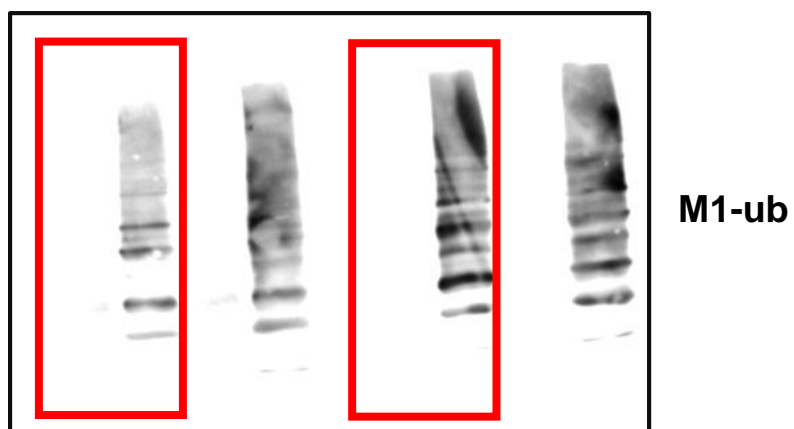

Supplement: Supplementary file 5 [file LSA-2022-01607_SdataF3.pdf]

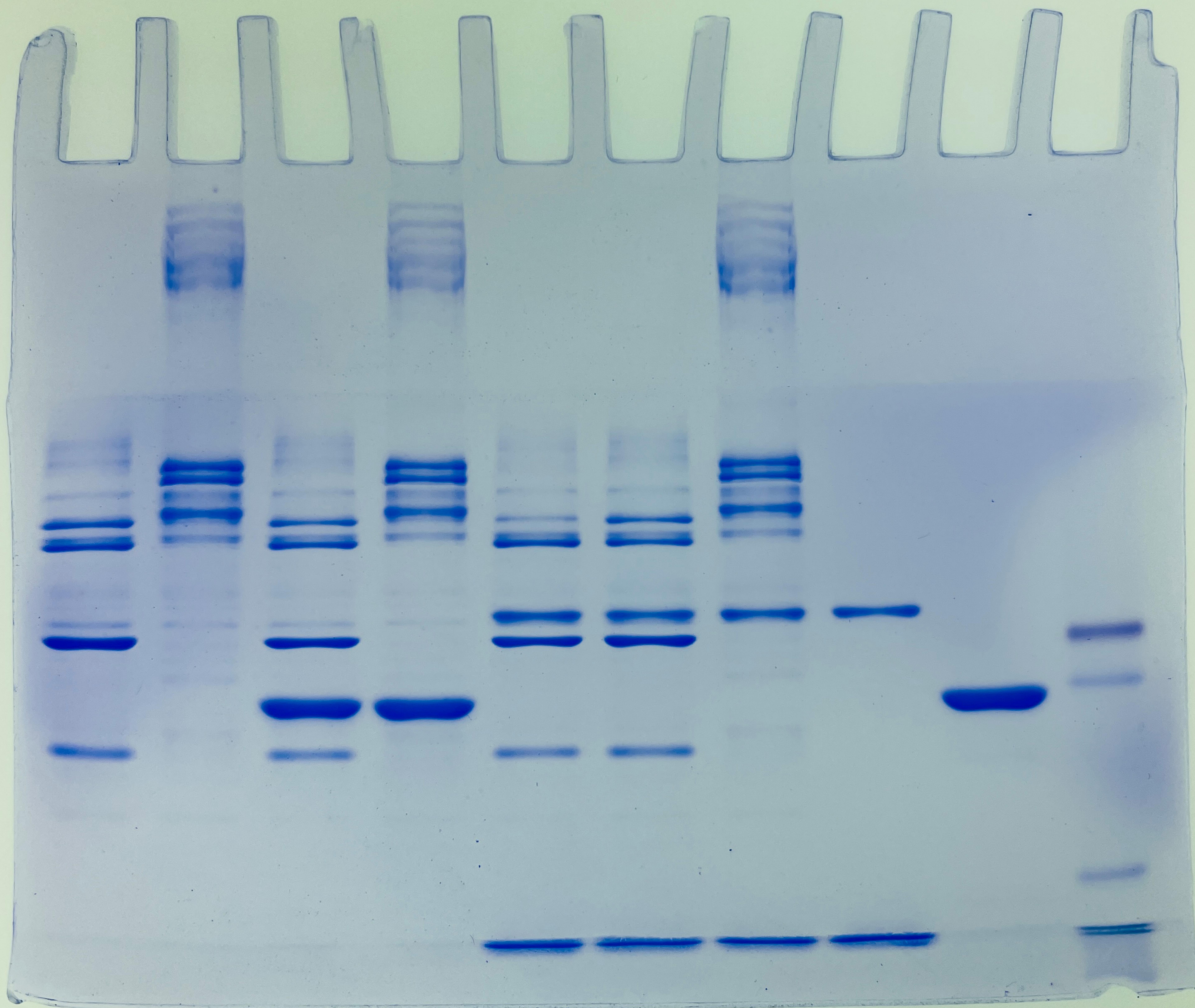

Supplement: Supplementary file 6 [file LSA-2022-01607_SdataF4.pdf]
